# Case report: Osimertinib administration during pregnancy in a woman with advanced EGFR-mutant non-small cell lung cancer

**DOI:** 10.3389/fonc.2023.1108124

**Published:** 2023-03-24

**Authors:** Pamela Soberanis Pina, Luis Lara-Mejía, Venecia Matias-Cruz, Feliciano Barrón, Andrés F. Cardona, Luis E. Raez, Eduardo Rios-Garcia, Oscar Arrieta

**Affiliations:** ^1^ Thoracic Oncology Unit, Department of Thoracic Oncology, Instituto Nacional de Cancerología (INCan), Mexico City, Mexico; ^2^ Direction of Research and Education, Luis Carlos Sarmiento Angulo Cancer Treatment and Research Center - CTIC, Bogotá, Colombia; ^3^ Thoracic Oncology Program, Memorial Cancer Institute/Florida Atlantic University, Miami, FL, United States

**Keywords:** case report, pregnancy, EGFR-TKI, lung adenocarcinoma, metastatic disease

## Abstract

Lung cancer (LC) is one of the most common causes of death worldwide. The identification of oncogene-addicted driving mutations suitable for targeted therapy has improved clinical outcomes in advanced diseases. Clinical trials, on the other hand, rarely involve vulnerable groups such as pregnant women. We report a 37-year-old woman with advanced non-small cell lung cancer (NSCLC) harboring an exon 19 deletion of *EGFR* treated with afatinib. After the initial treatment, the patient achieved a complete response and had an unplanned pregnancy. Targeted therapy was withheld during the first trimester and resumed with osimertinib in the second trimester in which the patient developed oligohydramnios and intrauterine growth restriction (IUGR) of the baby. Osimertinib was delayed at two different times during the third trimester with complete resolution of the oligohydramnios. The baby was born at 37.3 weeks of gestation (WOG) with no signs of congenital disorders. After delivery, the mother restarted osimertinib and maintained a complete response. This case suggests that osimertinib could be an acceptable option for tumor control during pregnancy in EGFR-mutant NSCLC. This information do not replace current recommendations for avoiding pregnancy and promoting contraceptive usage in patients receiving any cancer therapy.

## 1 Introduction

Lung cancer (LC) is the third most common cancer in women with a high mortality rate and represents the second cause of cancer death worldwide (1, 2). The incidence of LC during pregnancy is unknown, with less than 100 reported cases, and information regarding recommended therapy in this setting is even more scarce (3).

As genetic sequencing techniques are increasingly available, oncogenic driver mutations and other genomic alterations are frequently identified, guiding cancer therapy (4). In case of women of reproductive age, there are higher probabilities for harbored epidermal growth factor receptor (EGFR) mutations (5, 6).

There are limited data exploring the genomic characteristics and recommended cancer therapy in pregnant women with LC. Dagogo et al. reported a cohort of patients between 2009 and 2015, which included 160 women of reproductive age. Eight patients were diagnosed with LC during pregnancy, all harboring oncogenic driver mutations. Nevertheless, these patients never received any targeted therapy (7). This case describes the safety and efficacy of osimertinib treatment during pregnancy.

## 2 Case description

A 37-year-old non-smoker Hispanic woman without relevant medical background sought medical attention due to a 6-week history of cough, dyspnea, general weakness, and intermittent fever in March 2018. A computed tomography (CT) scan revealed a left lung solid lesion and ipsilateral pleural effusion. Histopathological analysis of the performed biopsy reported a poorly differentiated adenocarcinoma with a predominant micropapillary pattern, positive for cytokeratin 7, thyroid transcription factor-1, and napsin A. Pleural fluid cytological examination was positive for malignant cells. EGFR mutation (exon 19 deletion) was confirmed by polymerase chain reaction (PCR).

**Figure 1:**
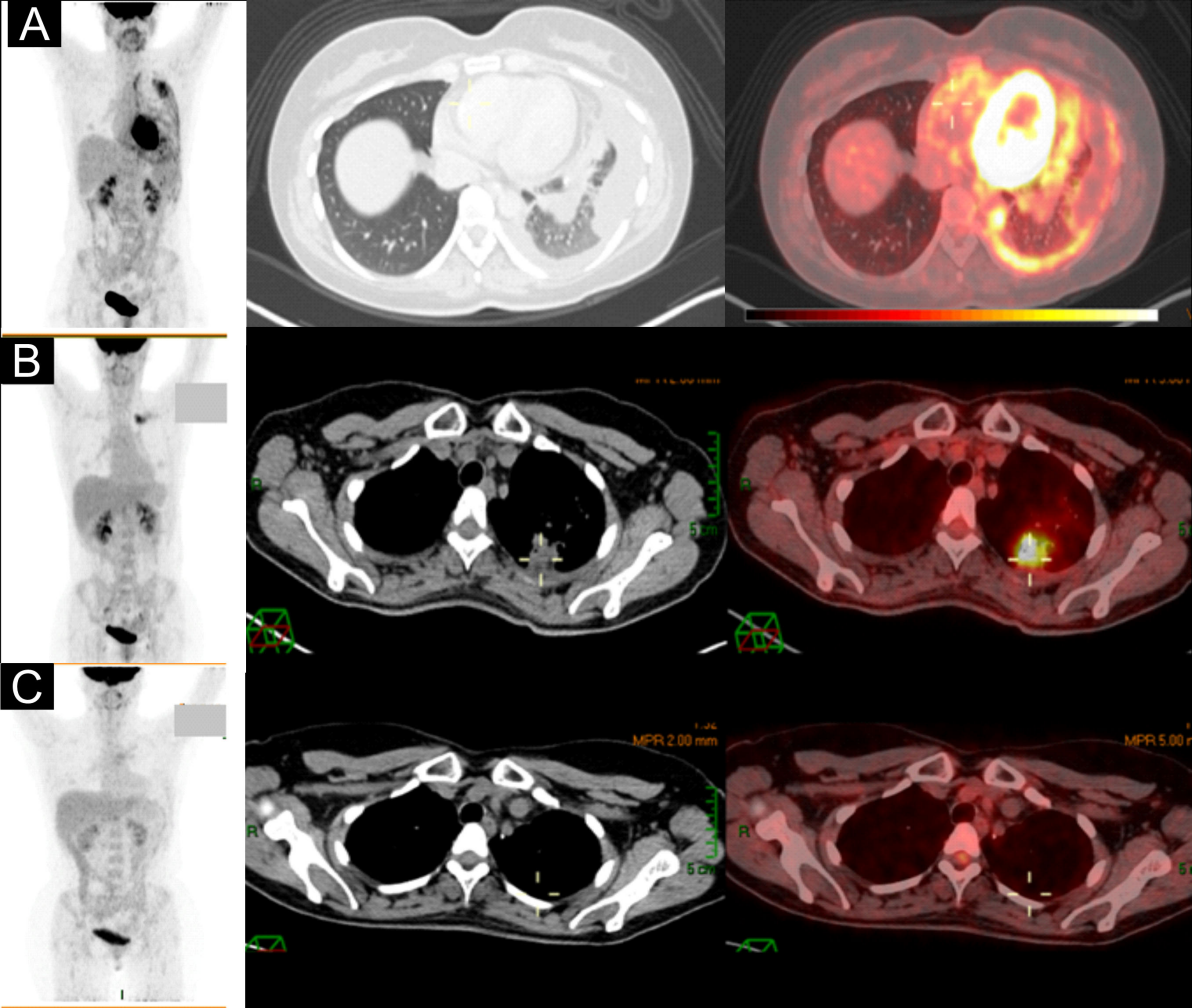
**(A)** Baseline PET-CT showed nodular thickening of the left parietal pleura, lung lesion in the left upper segment, and loculated pleural effusion. **(B)** PET-CT after SBRT revealed an increase in size and 18-FDG uptake. **(C)** PET-CT after 34 months of afatinib with complete response.

Positron emission tomography/computed tomography (PET-CT) showed nodular thickening of the left parietal pleura associated with pleural effusion and a dominant pulmonary lesion in the left upper lobe associated with an increased 18-FDG uptake (SUV_max_ 12.09). A secondary lesion was identified in the apicoposterior segment, also associated with an increased uptake (SUV_max_ 5.2) (**Figure 1A**). Brain magnetic resonance imaging (MRI) was negative for metastatic disease.

She started therapy with afatinib (40 mg per day) in April 2018. The first radiological evaluation at week 8 showed stable disease; however, the disease-related symptoms significantly improved. A second imaging evaluation in December 2018 exhibited a partial response, with residual disease in the apicoposterior segment, receiving stereotactic body radiation therapy (SBRT) as consolidative therapy. In July 2019, the patient experienced an oligoprogression within the radiation field, visualizing an enlargement and increased uptake in the follow-up PET/CT (**Figure 1B**). The patient underwent a left upper lobectomy and mediastinal lymphadenectomy achieving a complete response (CR) as the best outcome (**Figure 1C**). From August 2019 to January 2021, the patient continued afatinib until the 34th cycle maintaining CR. In January 2021, the patient became pregnant confirmed by hCG determination and transvaginal ultrasound showing the viability of the fetus. Once the pregnancy was documented, afatinib was discontinued. The fetus was exposed to afatinib for less than 6 weeks according to her last menstrual cycle. The mother decided to keep with the ongoing pregnancy after a discussion with the multidisciplinary tumor board (MTB) and institutional ethics committee.

**Figure 2:**
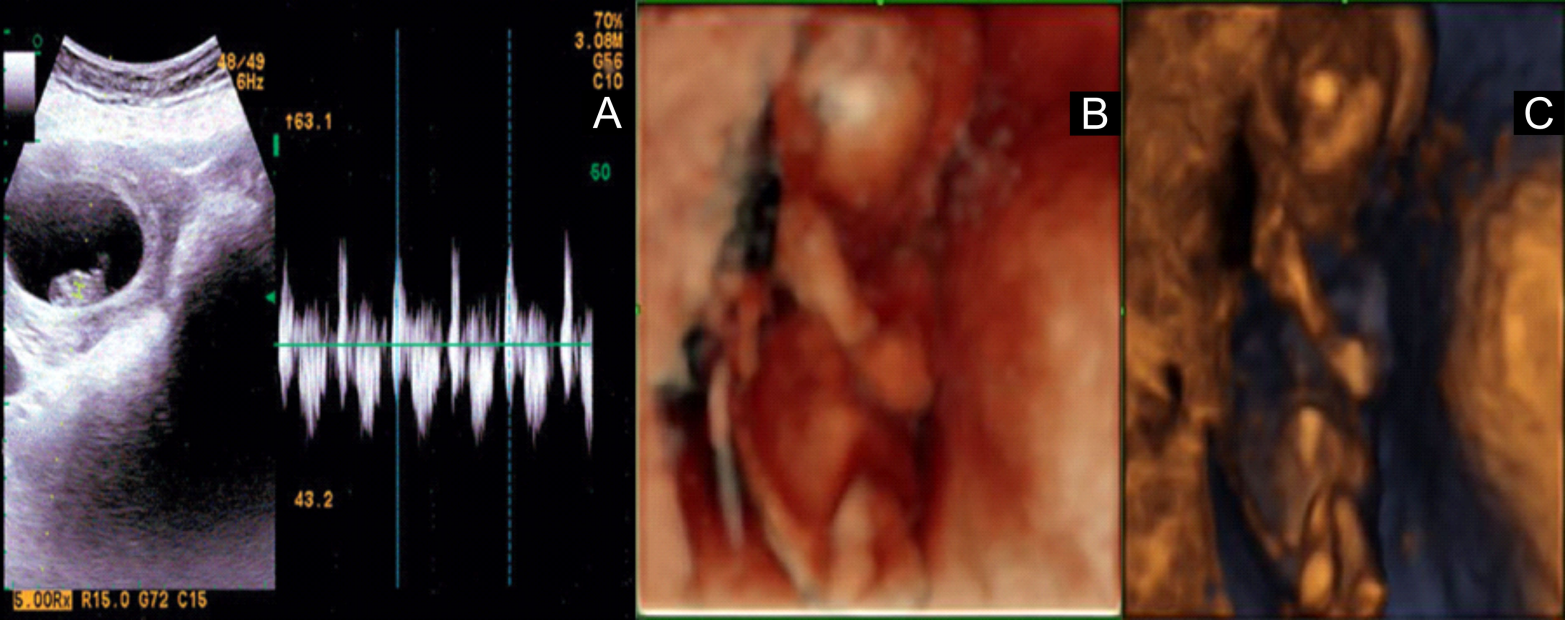
**(A)** First ultrasound at 10.5 weeks of gestation without alterations. **(B, C)** Normal structural ultrasound at 15.4 weeks of gestation (according to the last menstrual period). Average fetometry of 15.5 weeks for a weight of 124 g.

After counseling with the MTB, involving neonatologists, perinatologists, and medical oncologists, the chosen tyrosine kinase inhibitor (TKI) to proceed was osimertinib (80 mg per day) initiated at week 12, corresponding to the second trimester. Osimertinib was the preferred option based on the increased selectivity to EGFR binding pocket and was administered with acceptable tolerance during the second trimester (May 2021 to July 2021). The initial evaluation with a pelvic ultrasound reported no morphological alterations in the baby (**Figures 2A–C**). Nevertheless, at 28 weeks of gestation (WOG), severe oligohydramnios and intrauterine growth restriction (IUGR) (low fetal weight of 835 ± 107 g) were demonstrated in the ultrasound. As a result, osimertinib treatment was delayed and resumed 2 weeks later until the amniotic fluid was normalized and the fetal weight improved. At 32.4 WOG, the follow-up ultrasound revealed for the second time signs of oligohydramnios and reduced fetal weight (1,476 ± 145 g). At this time, the MTB decided to discontinue osimertinib until delivery.

At 37.3 WOG, an elective C-section was performed. The patient delivered a female baby weighing 2,466 g and measuring 48 cm. Apgar scores were 8 and 9 at 1 and 10 min, respectively. No congenital malformations or organic alterations were identified at childbirth. Screening for genetic and metabolic disorders ruled out any deficiency. The baby was discharged after 2 days in stable condition. No evidence of metastasis in the placenta or amniotic fluid was found. After delivery, the patient resumed osimertinib at the same dose. In her most recent PET/CT scan (February 2023), the patient continues without disease progression. At 16 months of age, the baby is healthy with no developmental alterations.

**Figure 3:**
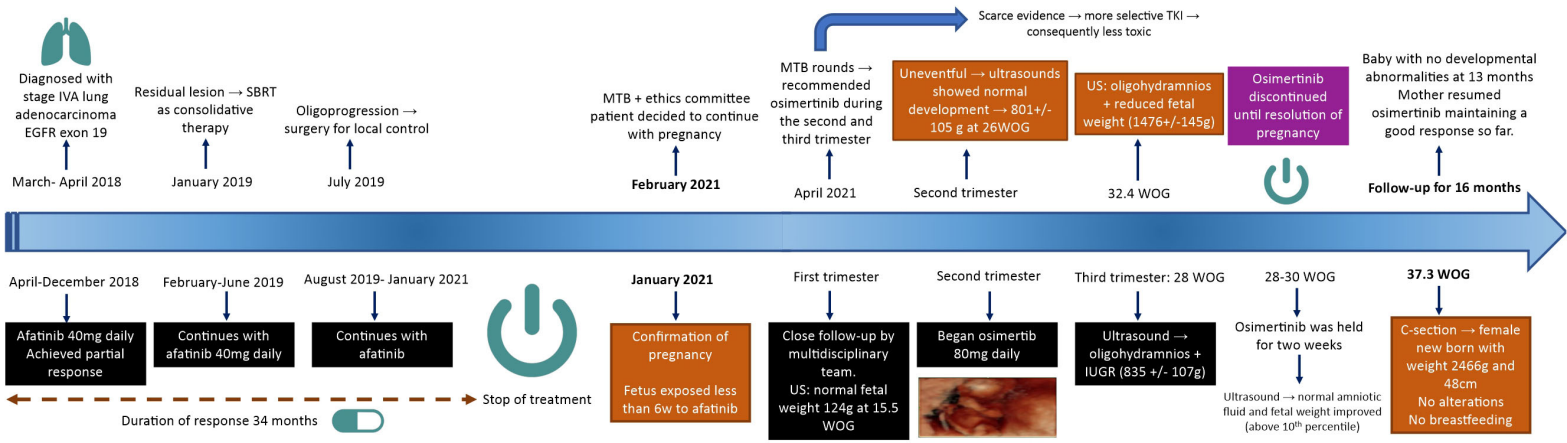
Timeline of clinical events from diagnosis to last follow-up appointment.

The clinical events described in this case are represented in a timeline in **Figure 3**.

## 3 Discussion

Targeted therapies are one of the biggest breakthroughs in cancer research. The identification of specific genomic alterations in different types of cancer has allowed the development of new drugs with increased specificity according to the mutations found (8). Oncogenic drivers in NSCLC such as EGFR mutations and anaplastic lymphoma kinase (ALK) rearrangements prevail in 10% to 20% and 2% to 7% of the patients, respectively. The incidence of these drivers is reported to be higher in Asia and Latin America compared with Caucasians (9). Particularly for EGFR-mutated tumors, several TKIs have been introduced to the current treatment landscape. Koojiman et al. showed the efficacy and potency of osimertinib in the presence of relevant EGFR mutations, including the ones conditioning resistance (p.L858R + p.T790M) (10). Other studies such as the network meta-analysis conducted by Qi et al. involving 4,389 patients showed that osimertinib has a longer progression-free survival (PFS) and a lower rate of adverse events than the combination of first-generation EGFR-TKIs + chemotherapy or second-generation EGFR-TKIs alone, showing the security profile of the selected therapy (11).

The available information concerning chemotherapy administration during pregnancy is limited in LC due to the low proportion of cases coexisting with pregnancy states. Therefore, evidence is scarcer regarding EGFR-TKI prescription during pregnancy. Previous studies have reported poor survival outcomes (less than 12 months after delivery) while using chemotherapy during pregnancy. In addition, there are safety concerns attributed to its potential harm to the fetus. Under anticancer therapy, the highest risk of teratogenicity in the fetus occurs during the first trimester. For this reason, almost all anticancer drug therapies are held until the second trimester reducing the risks of morphological alterations without compromising oncologic outcomes (3).

The EGFR pathway, with its respective ligands, is involved in various stages of embryonic development, placental function, early conception, and implantation phases (12). In preclinical models, EGFR inhibition can lead to fetal, perinatal, and postnatal complications associated with impaired epithelial development in several organs, including the kidneys. Interestingly, the kidneys express EGFR in collecting tubules in early and late gestational stages, while epidermal growth factor (EGF) and transforming growth factor (TGF) are prominently expressed in developing tubules and glomeruli. Amniotic fluid volume in the gestational sac is the product of a balance between fluid production and fluid elimination out of the sac. The kidney’s function plays a key role after week 20 of gestation, becoming the primary source of amniotic fluid production (13).

In the intrauterine stage, the EGFR pathway is also involved in renal electrolyte homeostasis and renal organogenesis. In this regard, preclinical studies have shown that erlotinib affected renal function through EGFR inhibition associated with decreased proliferation of proximal tubular cells, which in turn reflected the potential regulation of this pathway against kidney stress or damage (14). In concordance, ERBB2 receptors that belong to the same receptor family as EGFR (ERBB1) are also expressed in the fetal kidney, and trastuzumab, an anti-HER2 monoclonal antibody, has been potentially associated with fetal kidney impairment (15). This mechanism might be involved in altered amniotic fluid production under EGFR blockade. It could partially provide a reasonable explanation for the appearance of oligohydramnios even with a more selective EGFR-TKI like osimertinib. Notably, we do not discard the involvement of any other proliferative pathways with crosstalk activation *via* the EGFR pathway or other unknown mechanisms.

Likewise, the feto-maternal interface, especially the brush border, has a high density of EGFRs, a pathway that contributes to placental endocrine functions. The lack of EGFR or truncated forms of the receptor in placental tissues has been associated with intrauterine growth retardation, underscoring the importance of this pathway in the correct functioning and development of the fetoplacental unit (16).

In animal models, TKIs have been linked to maternal and fetal toxicity. Abortion might happen when standard regimens result in more than three times the plasma drug concentrations (3). For this reason, it is relevant to consider the intrinsic drug effects, length, and extent of exposure to counterbalance the potential harm with the benefits of target therapy. Erlotinib has demonstrated low-efficiency transplacental transfer, as well as gefitinib. It has been reported that there is almost 80% lower dose exposure of the fetus through placental filtration (17). A low efficiency of placental transfer diminishes fetal exposure and possible side effects. According to a previous report, the mean fetal transfer rates of gefitinib and erlotinib were 16.8% and 31.4%, respectively. Although both were safe, this evidence suggested that gefitinib could be a preferred agent in this context (18).

The latest evidence demonstrates that exposure to TKI may not be responsible for congenital abnormalities, even in the first trimester, although alterations such as IUGR and oligohydramnios can occur, with potential hazards for newborn babies. In a systematic review of the literature performed by Boudy and colleagues, 25 pregnant women with NSCLC and oncogenic driver genomic alterations were treated with an EGFR-TKI (*n* = 6) and an ALK inhibitor (*n* = 5). Most of them were diagnosed during pregnancy: one during the first trimester and six during the second trimester. Five of the patients were diagnosed with LC before conception and have received TKI as treatment during pregnancy. IUGR and oligohydramnios were observed but without detrimental effects on the fetus and no signs of neonatal toxicity (16). Thus, considering the clinical benefit provided by targeted therapy, EGFR-TKIs could be considered during pregnancy, at least after the second trimester. The possible side effects associated with TKIs are not fully understood and remain an unsolved problem that requires further investigations to clarify the pathogenesis that may induce changes in the amniotic fluid and fetal growth.

In 2011, Chaft et al. found that 23% of the population with EGFR-mutant lung cancer that discontinued TKI experienced a disease flare 8 days after drug interruption (19). In the case we presented, even though the patient persisted for several weeks without the EGFR-TKI, she maintained CR. Our hypothesis to explain this phenomenon is principally that the resection of the oligoprogression worked as a potential curative therapy. In addition, the long-term response to afatinib (34 cycles) when the average PFS is 11 months (20) suggests favorable tumor biology with a better prognosis. We cannot rule out immunological mechanisms driven by pregnancy as the ones described are not fully related to a systemic immunomodulator response but a local one in the feto-maternal interface (21, 22). Notably, the immune status is not constant during pregnancy and is considered an “immune clock of human pregnancy” allowing a potential effect on tumor response (23). Moreover, steroid hormones can be related more to a procarcinogenic activity due to the expression of estrogen receptors (ERs) and aromatase in the tumor microenvironment, conditioning possible tumor progression (24–26).

There is a paucity of data published about new-generation targeted therapy and immunotherapy given during pregnancy and the responses that were achieved. Previously, two case reports showed a near CR to CR of the disease in two patients that received trastuzumab and chemotherapy during pregnancy; nonetheless, no mechanisms have been clearly identified that could induce sustained CR (27, 28). In line with this, a report from last year by Evens et al. described the case of a patient with a history of classic Hodgkin lymphoma in CR that became pregnant. At 19 WOG, recurrence was documented and the patient was treated with chemotherapy for two cycles. Later, she developed worsening symptoms, and at 26 WOG, she was initiated on nivolumab. A partial response was achieved after 3 cycles at 32 WOG. She continued with the treatment for 6 total antenatal doses and had a vaginal delivery of a healthy male neonate. A restaging PET scan showed complete metabolic remission at 2 weeks postnatal (29). The role of these novel agents and the mechanisms involved in the response during pregnancy need to be elucidated. They might include crosstalk pathways given the similarity between the placenta and the tumor microenvironment, maternal immune tolerance, epigenetic regulation and expression of specific cancer biomarkers before pregnancy, and individual genetic landscape that could be associated with a favorable prognosis and long-term response (30). Altogether, while case reports have been published, translational and clinical studies are needed to provide crucial information in this context to guide individualized treatment during pregnancy.

## 4 Conclusion

We described a case of a 37-year-old woman with advanced NSCLC harboring an exon 19 deletion of *EGFR* with an unintended pregnancy, which after counseling received osimertinib during the second and third trimesters. The patient of this case report experienced oligohydramnios and IUGR; however, both sequelae were reversible with interruption of the medication and without compromising newborn integrity. Moreover, osimertinib was not associated with fetal loss. Although the information until this moment suggests that there is a low transplacental passage of TKIs, caution is still needed to draw firm conclusions. Further data, with longer follow-up and ideally robust evidence, are necessary to establish definitive recommendations in this context; until then, the more secure way is that every fertile woman with cancer should be advised and recommended to use contraceptives before starting active treatment. An updated report is planned in the future in which we plan to delve into the mechanisms of the response and look for minimal residual disease, ctDNA, and WGS if CR is maintained.

## Data availability statement

The original contributions presented in the study are included in the article/supplementary material. Further inquiries can be directed to the corresponding author.

## Ethics statement

The studies involving human participants were reviewed and approved by the Instituto Nacional de Cancerología Institutional Review Board and Ethics Committee. The patients/participants provided their written informed consent to participate in this study. Written informed consent was obtained from the individual for the publication of any potentially identifiable images or data included in this article.

## Author contributions

PSP contributed to the conceptualization, carried out the investigation, conceived and designed the experiments, and performed the formal analysis and writing—original draft of the manuscript. LL-M and ER-G carried out the investigation and contributed to the writing—review and editing, writing—original draft, methodology, supervision, and validation of results and figures. VM-C and FB contributed to the data curation, technical support for statistical and data analysis, and writing—review and editing of the manuscript. AC, LR, and OA contributed to the funding and project administration, conceptualization, and methodology. The original draft was prepared by PSP and OA. All authors contributed to the article and approved the submitted version.
